# Role of Carbon Dioxide, Ammonia, and Organic Acids in Buffering Atmospheric Acidity: The Distinct Contribution in Clouds and Aerosols

**DOI:** 10.1021/acs.est.2c09851

**Published:** 2023-08-21

**Authors:** Guangjie Zheng, Hang Su, Yafang Cheng

**Affiliations:** † Minerva Research Group, Max Planck Institute for Chemistry, Mainz 55128, Germany; ‡ Multiphase Chemistry Department, Max Planck Institute for Chemistry, Mainz 55128, Germany; § State Key Joint Laboratory of Environmental Simulation and Pollution Control, School of Environment, Tsinghua University, Beijing 100084, China; ∥ Chinese Academy of Sciences, Institute of Atmospheric Physics, Beijing 100029, China

**Keywords:** Acidity of aerosols and clouds, atmospheric multiphase reactions, organic acids, carbon dioxide (CO_2_), ammonia (NH_3_), multiphase buffering, cloud chemistry

## Abstract

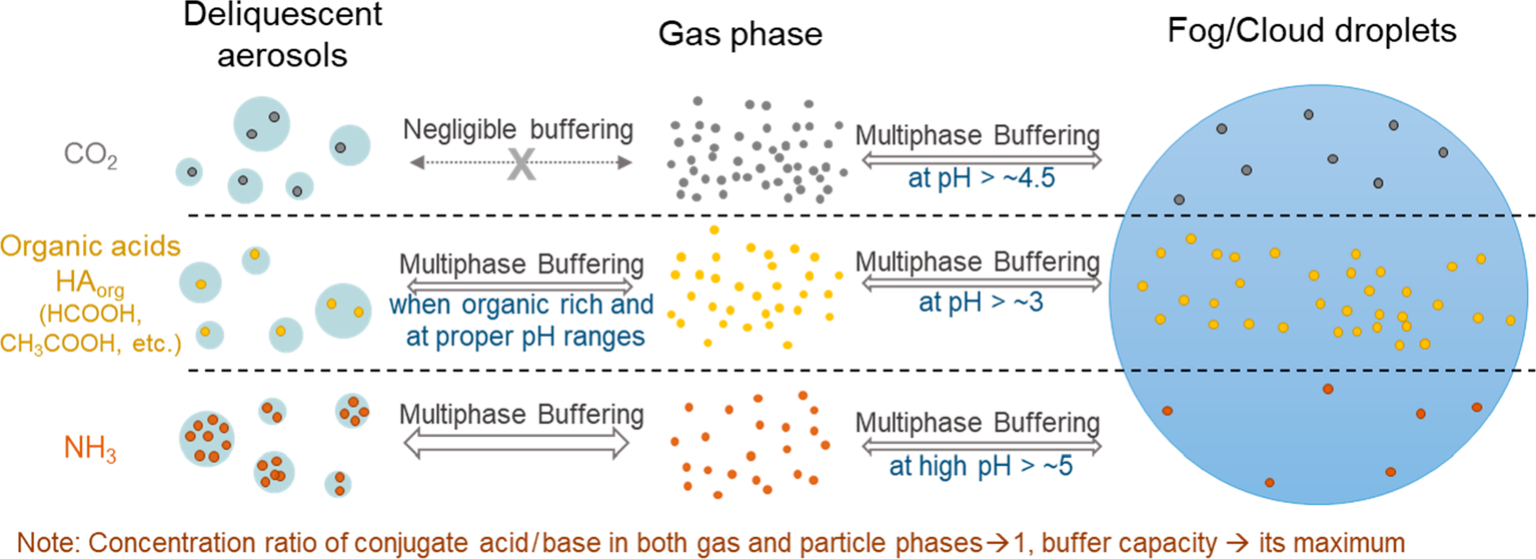

Acidity is one central parameter in atmospheric multiphase reactions, influencing aerosol formation and its effects on climate, health, and ecosystems. Weak acids and bases, mainly CO_2_, NH_3_, and organic acids, are long considered to play a role in regulating atmospheric acidity. However, unlike strong acids and bases, their importance and influencing mechanisms in a given aerosol or cloud droplet system remain to be clarified. Here, we investigate this issue with new insights provided by recent advances in the field, in particular, the multiphase buffer theory. We show that, in general, aerosol acidity is primarily buffered by NH_3_, with a negligible contribution from CO_2_ and a potential contribution from organic acids under certain conditions. For fogs, clouds, and rains, CO_2_, organic acids, and NH_3_ may all provide certain buffering under higher pH levels (pH > ∼4). Despite the 10^4^to 10^7^ lower abundance of NH_3_ and organic weak acids, their buffering effect can still be comparable to that of CO_2_. This is because the cloud pH is at the very far end of the CO_2_ multiphase buffering range. This Perspective highlights the need for more comprehensive field observations under different conditions and further studies in the interactions among organic acids, acidity, and cloud chemistry.

## Abstract 2

The different roles of carbon dioxide, organic acids, and ammonia in influencing the acidity of aerosols and clouds are illustrated.

## 1 Introduction

Atmospheric water, including aerosol water, fogs, clouds, rains, etc., are the major reaction sites of atmospheric multiphase chemistry, which is a major source of secondary species.^1−4^ Acidity of atmospheric water largely regulates the thermodynamics and chemical kinetics of atmospheric multiphase chemistry therein^4−7^ and therefore influences the effects of aerosols on health, ecosystem, and climate.^2,8−12^ Understanding the key influencing factors is thus crucial for accurate predictions of the acidity and efficiency of multiphase reactions.

Traditionally, acidity is thought to be determined by the relative abundances of atmospheric acidic versus alkaline species.^5,13−19^ Later studies, however, find that the acidity can vary much at given ratios of acids to bases,^20^ due to the large variations in the efficiency of these species in influencing the acidity, depending on their properties and environmental conditions. Here, the efficiency refers to the fraction of dissociated aqueous-phase anions/cations in atmospheric water that one species can contribute at given total (gas + particle phase) concentrations. For species associated with nonvolatile strong monoacids or bases (e.g., Na^+^, K^+^), the mechanism is the simplest, i.e., merely through neutralization reactions, and the efficiency can be considered as one. For nonvolatile weak acids or bases, they can influence the acidity through neutralization reactions and buffering effects. For semivolatile acids or bases, the mechanisms are more complex, where both gas–particle partitioning and aqueous-phase dissociation play a role in determining their efficiencies. Moreover, the nonideality, precipitation equilibrium, etc. would also influence the final acidity, especially in aerosol water.

Atmospheric weak acids and bases, mainly the CO_2_/HCO_3_^–^/CO_3_^2–^ system, organic acids, and ammonia, are long considered to play a role in regulating atmospheric acidity. Their quantified importance and major influencing mechanisms, however, seem confusing at a glance. For example, CO_2_ determines the pH of “pure” raindrops (∼5.6, namely, the pH when the water is in equilibrium with gas-phase CO_2_ mixing ratios of ∼350 ppm; see detailed processes in ref (2)), while its influence is often neglected in aerosol acidity calculations. In addition, while some studies^6,21−29^ suggest that carbonates and organic weak acids (e.g., formic acid, acetic acid, and oxalic acid) can “buffer” the aerosol acidity, the recently proposed multiphase buffer theory^7^ suggests that this effect is usually negligible compared with the ammonia multiphase buffering.^7,30^ In comparison, while the importance of ammonia in buffering the aerosols was well illustrated recently,^7^ its role in fogs/clouds is less understood. How and to what extent these species influence atmospheric acidity need to be clarified.

Here, we explored this issue with the recent research advances, especially the multiphase buffer theory.^7^ The importance and mechanisms of CO_2_, organic acids, and ammonia are discussed for different types of atmospheric water (aerosol water, fogs, and cloud droplets), and key uncertainties and future studies needed are also discussed.

## 2 Identifying Significant Contributors to the System Buffering Effect

**Figure 1:**
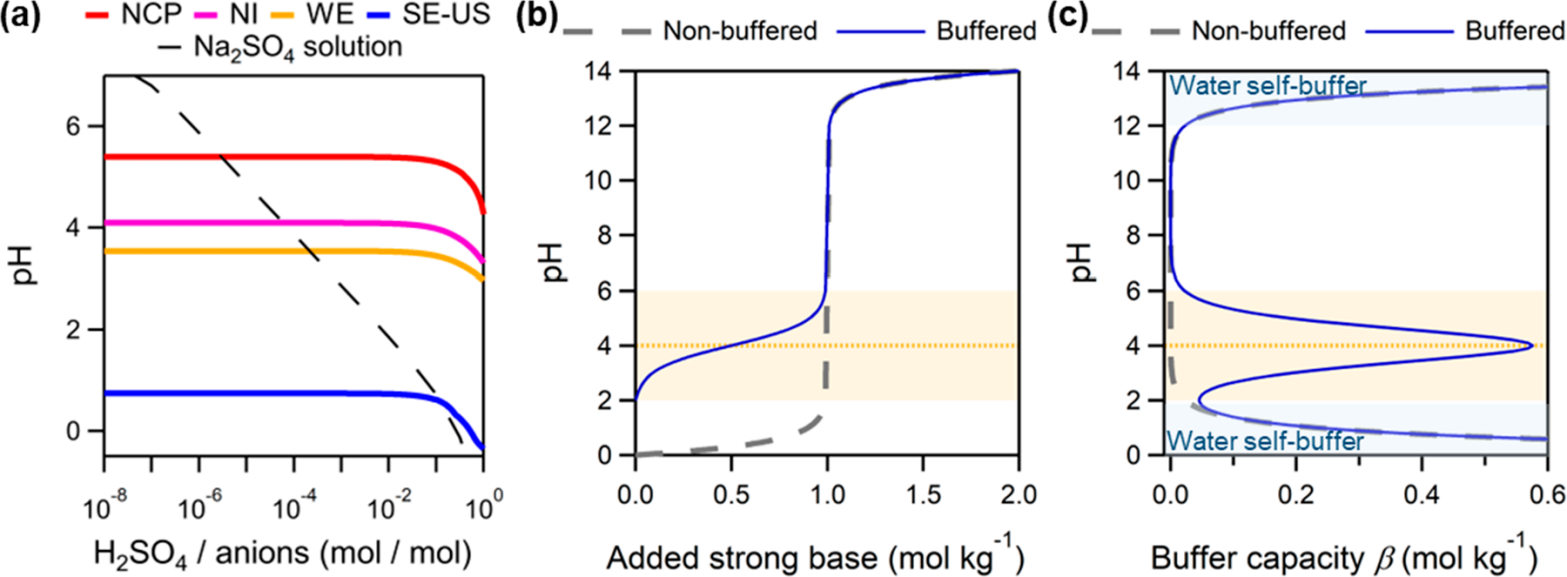
Influence of buffering effect on pH. (a) The buffering of aerosol pH observed worldwide. Taken from Figure S1 in ref (7) under the terms of AAAS Standard Author License to Publish. The *x*-axis is the molar ratio of sulfuric acid added to the anions initially present in the system. The “NCP”, “NI”, “WE”, and “SE-US” scenarios refer to winter North China Plain, northern India, western Europe, and summer southeastern U.S.A., respectively, and the response of the 2.5 mol kg^–1^ Na_2_SO_4_ aqueous solution is also shown for reference. See details in the Supporting Information of ref (7). (b, c) Comparison of the (b) pH and (c) buffering capacity during the titration process between buffered and nonbuffered bulk aqueous systems. Here, the titration process of adding a strong base (e.g., NaOH) into the solution with 1 mol kg^–1^ of strong acid like HCl (the nonbuffered system) or weak acid with a p*K*_a_ of 4 (the buffered system) is shown. The yellow shaded area indicates the buffering range of the weak acid.

The acidity of aerosols at various locations worldwide is buffered, and ammonia is proposed to be the dominant buffering species.^7,13^ As illustrated in Figure 1a, the aerosol pH shows little decrease until the added acid reaches a certain amount (molar ratios of equivalent added H^+^ reaches ∼20% of the initial amount of anions), which is in sharp contrast with the behavior of nonbuffered system like the Na_2_SO_4_ solutions.

The buffering effect is the process when the buffer agents, namely, the conjugate acid/base pairs that differ only by one proton, partially absorb the added H^+^ or OH^–^ through dissociation equilibrium. Take a weak acid HA with the acid dissociation constant *K*_a_ for illustration, upon the addition of strong acids/bases, it can buffer the pH changes through
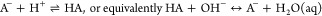
1awhich is a reversible reaction, and at equilibrium, the system should satisfy
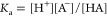
1b

Note that the buffering effect is significant only within the buffering range, i.e., a certain pH range around p*K*_a_. Outside these buffer pH ranges, the buffer agent exists predominantly either as [A^–^] or [HA], and the buffering effect is negligible, as detailed below.

### 2.1 Bulk Aqueous Solutions

The influence of buffering effects on acidity can be characterized by the buffering capacity β, which represents the resistance of pH changes upon addition of strong acids/bases, i.e.,
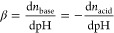
2awhere *n*_acid_ and *n*_base_ refer to the amount of added strong acids or bases.

For bulk aqueous systems, that is (see detailed derivation processes in refs (7 and 31))
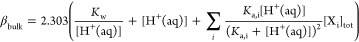
2bwhere *K*_w_ is the water dissociation constant, and *K*_a,*i*_ and [X_*i*_]_tot_ represent the acid dissociation constant and total molality of the buffering agent X_*i*_ (i.e., [HA]+[A^–^] for weak acids), respectively. Note that for nonbuffered aerosol systems, X_i_ = 0, and β is
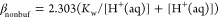
3where the remaining terms represent the water self-buffering effect.^7^

Figure 1b shows the difference in the titration process between a buffered and a nonbuffered bulk aqueous system. For illustration, here we show the titration curve of adding a strong base (e.g., NaOH) into the solution with 1 mol kg^–1^ of (i) strong acid like HCl (the nonbuffered system, dashed gray line) or (ii) weak acid with a p*K*_a_ of 4 (the buffered system, blue line). For the nonbuffered strong acid solution, the pH changes abruptly from ∼0 to ∼14 around the midpoint, i.e., when the added strong base equals the existing strong acid with the solution pH 7. For the buffered system with the weak acid, however, the pH changed slowly around the p*K*_a_ of 4 upon the addition of strong base within the buffering pH ranges, indicating a strong buffering effect. Correspondingly, the β of the buffered system (β_bulk_) differs much with that of the nonbuffered system (β_nonbuf_) in this pH range (Figure 1c). When the added strong base is too much and the pH is elevated outside the buffering pH range (roughly pH above 6), the titration pH curve of the buffered system is roughly the same as the nonbuffered system (Figure 1b), indicating a negligible buffering effect. Correspondingly, β_bulk_ and β_nonbuf_ differ little in this pH range (β_bulk_ – β_nonbuf_ < 0.02 mol kg^–1^; Figure 1c), indicating the negligible buffering effect.

### Importance of Buffering Effect of a Given Buffer Agent

Based on the analysis above, we can see that the contribution of a potential buffer agent to the system buffering effect is
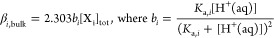
4

We propose that the buffering effect of a potential buffer agent at a given system pH can be treated as negligible when
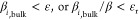
5where ε and ε_r_ are both arbitrarily selected small numbers close to zero and represent the minimum absolute and relative buffering capacity of interest, respectively. When either of the above 2 criteria is met, the buffering capacity provided by buffer agent *i* would be too small, so that the difference in pH responses upon addition of strong acids/bases with/without this buffer agent is hardly discernible. That is, the buffering effect of agent *i* is negligible. In the case shown in Figure 1, the criterion of ε of 0.02 mol kg^–1^ is applied.

According to eq 4, the influencing factors of *β*_*i*__, bulk_ is the total amount of buffering agents [X_*i*_]_tot_ and the coefficient *b*_*i*_, and *b*_i_ is determined by the difference between the system pH and the p*K*_a,*i*_ (SI Text S1; Table S1). When pH and p*K*_a,*i*_ differ too much (e.g., 6), the *b*_*i*_ is so small (e.g., 1.0 × 10^–6^) that the β_*i*_ is significant only when [X_*i*_]_tot_ is extremely large (e.g., on the order of 10^5^ mol kg^–1^ for the ε of 0.1 mol kg^–1^). Accordingly, the more abundant the buffering agent is, the larger buffer pH ranges it would influence.

### 2.2 Multiphase Systems

The above analysis can be easily applied to multiphase systems like aerosols, when we replace β_bulk_ in the bulk aqueous solutions (eq 2a) by β_mp_ in the multiphase system^1,21^
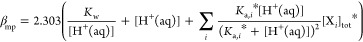
6awhere *K*_a,i_* is the effective acid dissociation constant, and [X_*i*_]_tot_* is total equivalent molality of X_*i*_ including those that exist in the gas phase, as the gas–particle partitioning also plays a role. For a semivolatile acid HA and a semivolatile base BOH, the *K*_a_* are, respectively,
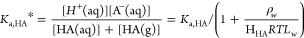
6b
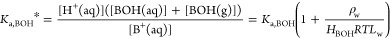
6cwhere *H*_X_ is Henry’s constant (i.e., gas–particle partitioning constant) of species X in mol L^–1^ atm^–1^, *L*_w_ is the liquid water content in (g water)/(m^3^ air), ρ_w_ is the liquid water density (∼10^6^ g m^–3^), *R* is the gas constant (8.205 × 10^–2^ atm L mol^–1^ K^–1^), and *T* is the absolute temperature in K.

Similarly with that in the bulk aqueous solutions, β_*i*_ in multiphase systems is
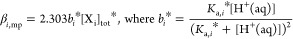
7and is determined by *b*_i_* and [X_*i*_]_tot_*, where *b*_i_* depends on |pH–p*K*_a,i_*| (SI Text S1; Table S1), while p*K*_a,i_* depends further on *K*_a,*i*_, *H*_*i*_, *L*_w_ and *T* (eq 6b). The *L*_w_ of aerosols (i.e., aerosol water contents) typically varies between 10^–6^ and 5 × 10^–4^ g m^–3^, while for clouds and fogs it can range between 0.05 and 3 g m^–3^ but is usually from 0.1 to 0.3 g m^–3^ (ref (2)). Note that the *L*_w_ values of typical raindrops are usually on the same order of precipitating clouds.^32,33^ Even for severe storms, the *L*_w_ values are <14 g m^–3^ (ref (34)). Therefore, here we consider the *L*_w_ range of interest for atmospheric water as from 10^–6^ to 14 g m^–3^. Note that fogs, rains, and storms can all be viewed as a special type of activated water droplet.

Similar to the bulk aqueous phase, the importance of the multiphase buffering effect can be judged by eq 5. In this study, we arbitrarily set ε_r_ as 1%, and ε as the changes in particle-phase anion/cation molality corresponding to 0.001 μmol m^–3^ of changes in atmospheric concentrations, i.e.,
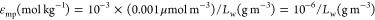
8where 10^–3^ is the unit converter from (μmol g^–1^) to (mol kg^–1^). This is roughly the smallest measurement uncertainty of typical atmospheric species (e.g., 0.05 μg m^–3^ of sulfate or 0.02 μg m^–3^ of ammonium) and would represent the perturbation of interest for most studies. The ε_mp_ thus represents the minimum buffer capacity that would provide this kind of minimum resistance of interest. Therefore, eq 5 can be rewritten as
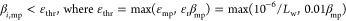
9

## 3 Role of CO_2_ and NH_3_ Systems

### 3.1 NH_3_/NH_4_^+^ Buffer Pair

**Figure 2:**
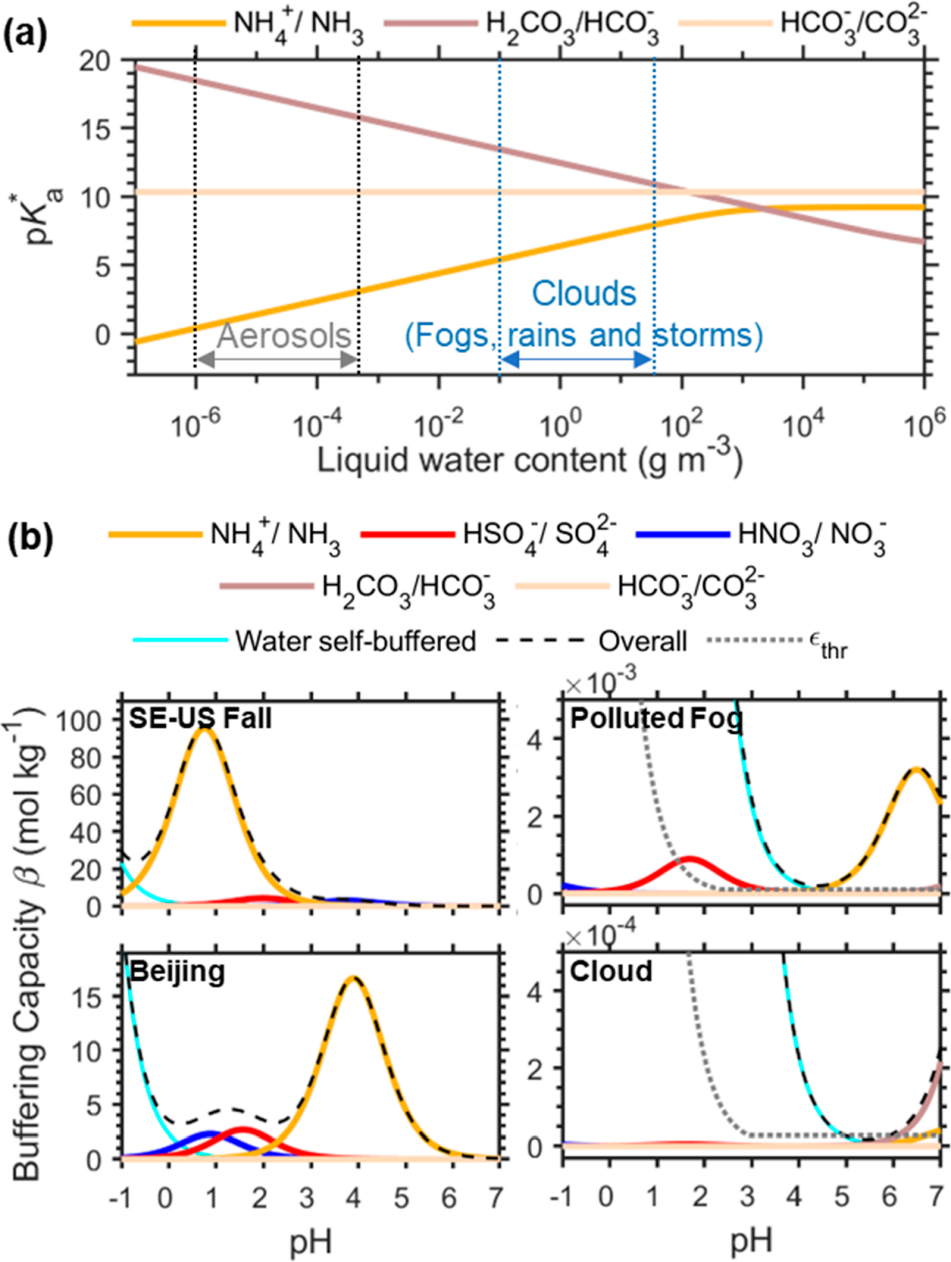
Importance of inorganic carbon systems in buffering the atmospheric water. Here, we assume a constant CO_2_ of 410 ppm. (a) Variation of the p*K*_a_* of H_2_CO_3_/HCO_3_^–^, HCO_3_^–^/CO_3_^2–^ in comparison with that of NH_4_^+^/NH_3_ with liquid water content *L*_w_ at 298 K. (b) The buffering capacity curves under four representative scenarios: the organic-rich clean southeastern U.S.A. aerosols in fall (SE-US Fall),^37^ the more polluted winter aerosols in Beijing,^38^ the polluted fog in San Joaquin Valley, California,^39−41^ and a cloud case.^42^ See detailed scenario settings in Table S2.

While ammonia is a weak base, it works mostly like a weak acid in the atmospheric multiphase system,^7,35^ with the p*K*_a_* increasing from ∼0.4 to ∼7.5 at 298 K in the *L*_w_ range of interest (Figure 2a, orange line). This agrees well with the typical pH ranges of atmospheric water of <7 (ref (5)). With the high abundances (i.e., high [X_*i*_]_tot_*) and the general agreement between p*K*_a_* and pH (i.e., high *b*_i_*), the NH_3_/NH_4_^+^ pair appears to be the dominant buffering species of aerosols for most populated continental areas (Figure 2b), where the aerosol pH usually varies around the p*K*_a_* of NH_3_. This has been well illustrated elsewhere (refs (7, 30, and 36)).

For fogs and clouds, the abundances of ammonia are less studied, but are usually considered as lower than those in surface aerosols.^2^ Meanwhile, the p*K*_a_* of ammonia is elevated considering the higher *L*_w_ range of fogs/clouds, which is 5.4–7.5 at 298 K and even higher (6.7–8.9) at 273 K (Figure S1). Therefore, its buffering capacity is much lower than in aerosols, which gradually exceeds ε_thr_ only at higher pH levels (> ∼4.5 for the “polluted fog” case and > ∼5.5 for the “cloud” case in Figure 2b). Nevertheless, it can still serve as the dominant buffering agent for the polluted conditions with high ammonia concentrations and high pH of 6–7, like the fogs observed in California’s San Joaquin Valley^39−41,43,44^ (i.e., “polluted fog” case in Figure 2b) and Italy’s Po Valley.^45−47^ For acidified clouds/acid rains with lower pH of ∼4 (ref (5)), the overall importance of ammonia buffering can be much lower or sometimes negligible (e.g., Figure 2b, “cloud” case). More observations and further studies are needed to illustrate the frequency of occurrence and situations when it is important.

### 3.2 CO_2_/HCO_3_^–^ Buffer Pair

As carbonic acid is a weak acid with a p*K*_a_ of ∼6.4 and giving the high mixing ratios of CO_2_ in the atmosphere, it was considered to strongly “buffer” the atmospheric water.^21−26^ Especially, the pH of “pure” rainwater of ∼5.6 is determined by CO_2_, which just falls into the buffering pH ranges of H_2_CO_3_ (6.4 ± 1), seemingly to support the strong buffering effect of CO_2_ on rain.

Analysis based on multiphase buffer theory, however, indicates that the buffering effect of CO_2_ is negligible for aerosols and limited for fog, cloud, or rain (Figure 2). In the *L*_w_ range of aerosols (10^–6^ to 5 × 10^–4^ g m^–3^), the corresponding p*K*_a_* of CO_2_/HCO_3_^–^ at 298 K is 15.8–18.4 (Figure 2a, gray line) with little influence from temperature (Figure S1). This is much larger than the typical pH ranges of aerosols. Even if we assume a fresh sea salt or dust aerosol with pH of 7–8, the |pH–p*K*_a_*| gap is still over ∼8, which corresponds to a *b*_i_* of <1 × 10^–8^ and renders β_mp,CO2_ negligible (i.e., much smaller than ε_mp_) even considering its high abundance (Figure 2b). The strong nonideality in aerosol water may influence the pH and p*K*_a_* by ∼1 unit,^36^ which still corresponds to a small *b*_i_* of <1 × 10^–7^.

For fogs, clouds, and rains, the higher *L*_w_ range decreases the p*K*_a_* of CO_2_/HCO_3_^–^ to around 11–13 (Figure 2a, Figure S1). Although the |pH–p*K*_a_*| gap is still large (>4), the corresponding *b*_i_* of <1 × 10^–4^ may be compensated by its high abundances when the cloud pH is higher. As shown in the example cloud case^42^ (Figure 2b, “cloud” case), β_mp,CO2_ becomes important (i.e., exceeds ε_mp_) when pH is over 5. This is consistent with the finding that for some fog samples in California’s San Joaquin Valley, the measured internal buffering intensity can be nearly accounted for by the carbonate system, especially in the pH ranges of 5–6.5 (ref (22)). Nevertheless, the buffering effect of CO_2_ was only comparable to that of ammonia, despite the >10^5^ higher abundances of total CO_2_ than total ammonia (Table S2).

The “pure” raindrop pH of ∼5.6 is derived when the water is in equilibrium with gas-phase CO_2_ mixing ratios of ∼350 ppm (see detailed processes in ref (2)). The role of CO_2_ during this process, however, is actually acidification, where the semivolatile carbonic acid acidified the pure water. This should not be confused with buffering, which is associated with the sensitivity of the system pH to the uptake of additional acids/bases. The limited buffering effect of CO_2_ can also explain the formation of acid rain and the rain pH in remote background areas. For example, the rain pH in remote background areas is typically 4–5 (refs (48 and 49)), which is lower than the pH when the water is in equilibrium with gas phase CO_2_ (i.e., the “pure” raindrop pH) of ∼5.6. This is usually attributed to the acidification by the naturally produced sulfate and weak organic acids.^48,49^ However, based on the traditional buffer theories of bulk aqueous solutions, the “pure” raindrop pH of ∼5.6 just falls in the pH range when the buffering effect of the CO_2_ is the strongest (6.4 ± 1). In this case, it was hard to imagine that the high peak β associated with the abundant CO_2_ (∼410 ppm) could be readily overcome by the trace amount of naturally produced acids so that the rain pH is acidified from ∼5.6 to 4–5 (refs (48 and 49)). Similarly, the acid rain is usually attributed to the anthropogenic acid gases like SO_2_ or NO_x._ However, these acid gases are typically smaller than several tens of parts per billion, which is from 10^4^ to 10^5^ lower than that of CO_2_ and would hardly compete with the high peak β of CO_2_ to acidify the water substantially. Based on the multiphase theory, however, we can see that the high abundance of CO_2_ is largely undermined by the large |pH–p*K*_a_*| gap; thus, its β_mp_ is only negligible to limited in the rain pH ranges and can be readily overcome by the acidification of trace acidic gases. See more detailed discussions in the illustrative case studies in SI Section S2 and Figure S2.

### 3.3 HCO_3_^–^/CO_3_^2–^ Buffer Pair

The HCO_3_^–^/CO_3_^2–^ buffer pair is nonvolatile, and its p*K*_a_ is always kept at ∼10. Following the above analysis procedures, we can conclude that its buffering effect is negligible in all atmospheric water, from aerosol water to clouds or rains. In comparison, both the bicarbonate and the carbonate salts, widely existing in dusts, etc., can neutralize the acids and therefore decrease the acidity. This neutralization process is sometimes termed as “buffering” to indicate that the existence of dusts can alleviate acidifications.^23,50−57^ We call for the use of “neutralization” instead of “buffering” for this process to avoid confusion in the future.

## 4 Role of Organic Acids

### 4.1 Influencing Factors of the Contributions to Buffering Effects

The organic acids, mostly carboxylic acids, are found to contribute significantly to both the free acidity (i.e., amount of dissociated acids) and total acidity (i.e., amount of acids in both dissociated and undissociated form)^58,59^ of precipitations and therefore acid rains.^60,61^ Especially in remote areas, their contributions can be dominant (up to 80%)^49,62−65^ and are still increasing.^66^ These indicate important contributions of organic acids to acidity through neutralization reactions (i.e., acidification). While the acidity of samples collected in bulk solutions (collected fogs, rains, or water extracts of aerosols), as characterized by indicators like free acidity of precipitations, is of interest in terms of the acidification of ecosystems, it is the in situ acidity that matters during the atmospheric chemical processes. The importance and mechanisms of organic acids in influencing the in situ acidity of atmospheric water, however, are still under debate. Some studies suggested a large potential of organic acids to buffer the pH of aerosols and fogs and therefore influence the atmospheric processing,^22,27−29^ while some others suggested negligible buffering effects.^67,68^

Here, we examined the potential contribution of organic acids to system buffering effects with the methods outlined in Section 2. One major concern is whether the organic acids can buffer in the typical pH ranges of atmospheric water, i.e., the influence of |pH–p*K*_a,i_*|. Based on eq 7b, we see the equivalent acid dissociation constant in multiphase system, *K*_a_*, differs from *K*_a_ by
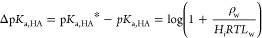
10which depends on the *H*_*i*_ and *L*_w_ at a given temperature (Figure S3). When ρ_w_(*H*_*i*_*RTL*_w_)^−1^ ≪1, Δp*K*_a_ ≈ 0, and *K*_a_* is roughly the same as *K*_a_. At 298 K, this is roughly when the product of *H*_*i*_ and *L*_w_ is over 5 × 10^5^ (mol kg^–1^ atm^–1^)(g m^–3^). That is, if one species is more soluble (with higher *H*_*i*_), its *K*_a_* will approach *K*_a_ and become insensitive to *L*_w_ at lower *L*_w_ levels.

Table S3 lists the thermodynamic properties of commonly observed water-soluble organic acids. For these organic acids, the p*K*_a_ mostly ranges 3–5, and the *H*_*i*_ mostly ranges from 10^3^ to 10^12^ mol kg^–1^ atm^–1^ (Table S3). Therefore, for most of these species, the Δp*K*_a_ will be <3 in clouds (*L*_w_ > 0.1 g m^–3^),and thus would be buffering at the appropriate pH ranges of <7. For aerosol water, however, the potential contribution to system buffering would differ greatly with the p*K*_a_ and *H*_*i*_.

Another concern is the influence of species abundances, i.e., the influence of [X_*i*_]_tot_. However, full-spectrum measurements of all atmospheric organic acids in both gas and particle phases are unlikely considering their wide variety and low concentrations of some certain species. Therefore, equivalent concentrations of representative species may provide a good first-order estimate. As formic, acetic, and oxalic acids are the most abundant and most widely measured organic acids,^60,62,69−73^ they can serve as good representative species, as detailed below.

### 4.2 Contribution to Buffering Effects in Aerosols

**Figure 3:**
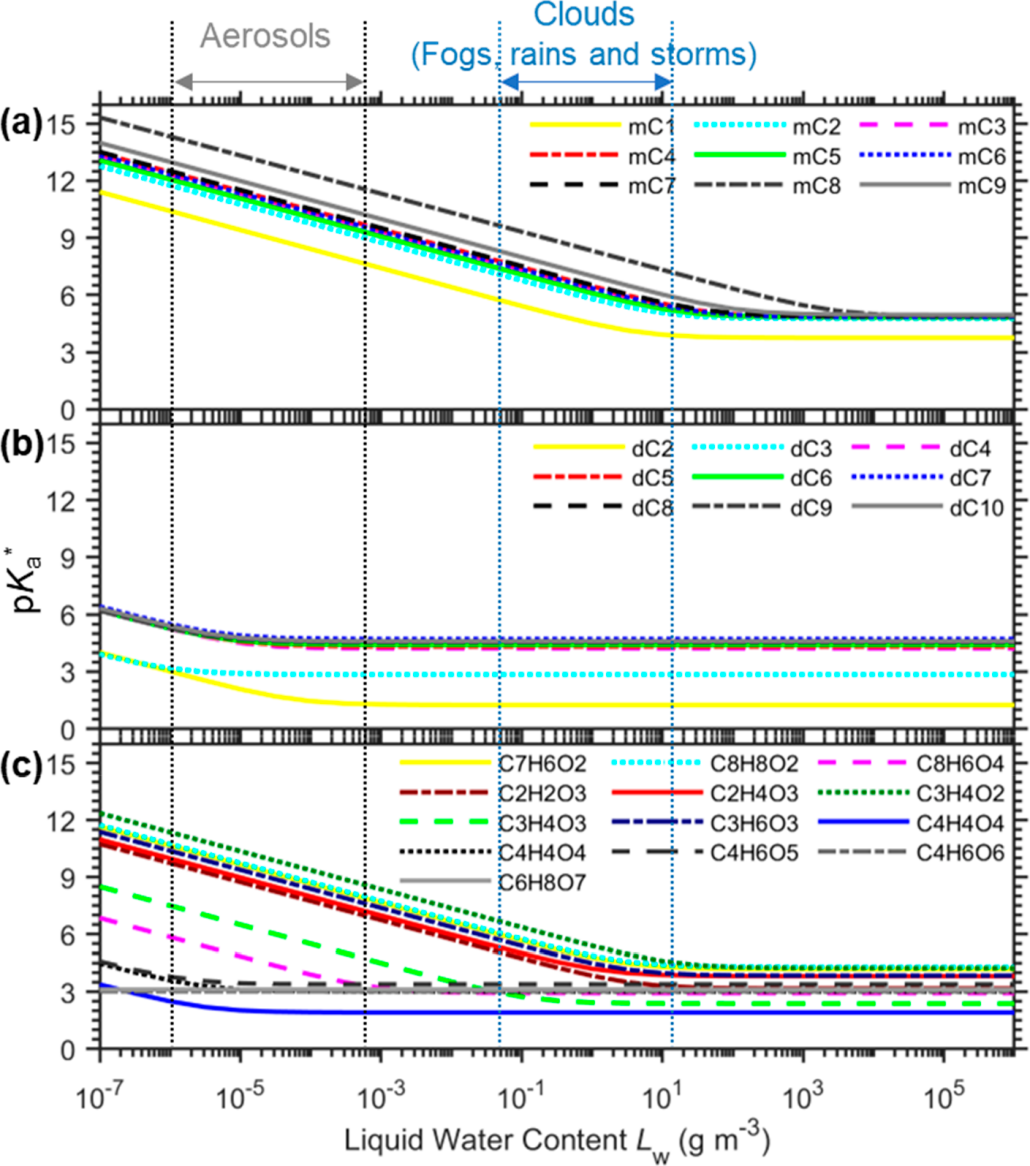
Variation of the equivalent multiphase acid dissociation constant *K*_a_* with liquid water content *L*_w_ for commonly observed organic acids in the atmospheric at 298 K. (a) C1–C9 *n*-alkanoic monocarboxylic acids, (b) C2–C9 aliphatic dicarboxylic acids (dC2–dC9), and (c) other acids. See the explanations of the abbreviations in Table S3.

Figure 3 shows the p*K*_a_* of atmospheric organic acids, as listed in Table S3. At the typical *L*_w_ range of aerosols, the p*K*_a_* of most *n*-alkanoic monocarboxylic acids are too high (> ∼8; Figure 3a), and their buffering effects are negligible due to the large pH–p*K*_a,i_* gap. Correspondingly, these acids are found to reside mostly in the gas phase in the absence of fogs/clouds.^37,38,76,77^ In comparison, the C2–C9 aliphatic dicarboxylic acids and some other acids (Figure 3b, c) are with the p*K*_a,i_* values of 2–6 and may buffer the aerosols. These potential buffering species are flagged in Table S3 (see the column “potential aerosol buffers”).

**Figure 4:**
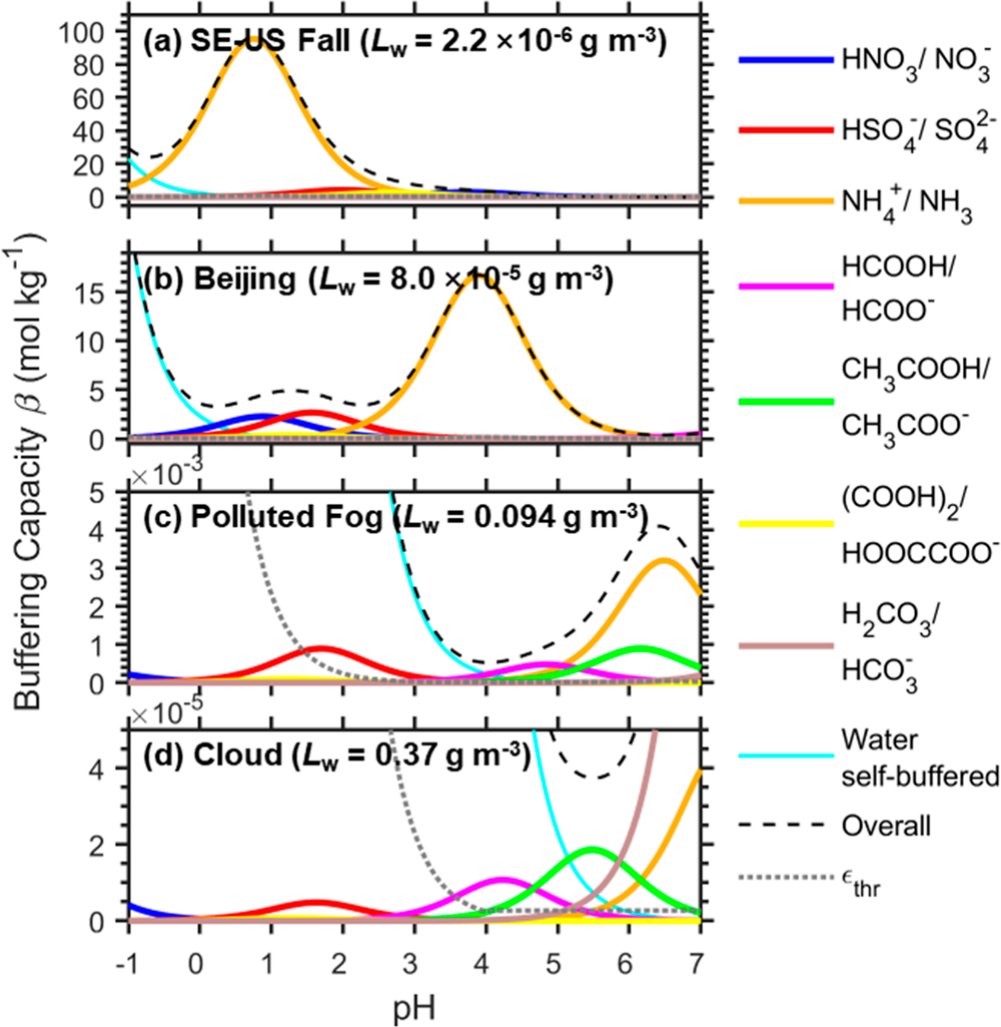
Buffering effects of the most abundant atmospheric organic acids of formic, acetic, and oxalic acids under (a) an agriculturally intensive region in the southeastern U.S.A. in fall 2016, which represents an organic-rich environment,^37^ (b) a more polluted urban area in Beijing in winter 2002, which is less organic rich, (c) the polluted fog in San Joaquin Valley, California,^39−41^ and (d) a cloud event (event #1) observed at the summit of the Puy de Dôme, France, in winter 2001.^42^ See detailed scenario settings in Table S2. Note that in aerosol cases of (a) and (b) the concentration of total oxalate acid is enhanced by 10 times to provide an upper-limit estimate of all organic acids that would potentially buffer in aerosols.

Among the above potential buffering species for aerosols, oxalates are usually the most abundant and typically account for 30%–80% of all detectable particle-phase organic acids.^78−80^ For an upper-limit estimate, we assume that all of these potential buffering organic acids are buffering at the same pH range with a total abundance of 10 times that of total oxalates. Even so, the total concentrations of these organic acids are much lower than the inorganic buffering pairs like NH_4_^+^/NH_3_ and are negligible in urban areas like Beijing (Figure 4b). Even in the organic-dominated areas like the agriculturally intensive rural southeastern U.S.A. site (Figure 4a), they may have a certain buffering effect only in the pH ranges when the contribution of NH_4_^+^/NH_3_ is negligible (i.e., outside the ammonia-buffered pH ranges).

### 4.3 Contribution to Buffering Effects in Fogs, Clouds, and Rains

For fogs and clouds, the aqueous-phase molalities of organic acids can be much lower than in aerosols due to the dilution of the much higher *L*_w_. Therefore, the buffering capacity of organic acids with a p*K*_a,i_* of <3 could hardly compete with the water self-buffering effect (Figure 4c) and can be negligible. This would exclude some species that have a potential contribution to buffering effects in aerosols (see Table S3, column “potential cloud buffers”). In comparison, while most of the monocarboxylic acids cannot buffer in aerosols, they have p*K*_a,i_* values of 4–7 in the *L*_w_ range of fogs and clouds (Figure 3a) and may contribute the system buffering (Figure 4c).

Measurements of chemical compositions including organic acids in both gas and particle phases of clouds/fogs are scarce. Figure 4(c) and (d) shows one polluted fog case in California’s San Joaquin Valley^39−41^ and one cloud event at the summit of the Puy de Dôme, France,^42^ while the situation may differ further in other places. As shown in Figure 4(c) and (d), the inorganic acids of HSO_4_^–^ and HNO_3_ are buffering at too low pH levels (<3), and their contributions to the buffering capacity are mostly below 1% that of a water self-buffering event (ε_thr_; gray dotted line in Figure 4). The contribution of oxalate acid buffering can be negligible due to both the low concentrations and the low buffering pH ranges. In comparison, HCOOH and CH_3_COOH pairs can provide certain buffering effects at higher pH ranges of > ∼3.5. The HCOOH pair can even serve as the dominant buffering species in the pH ranges of 4–5 for the polluted fog case (Figure 4c), while the CH_3_COOH pair can dominate the buffering in the pH ranges of 5.1–5.9 for the cloud case (Figure 4d). In scenarios when the organic acids are more abundant (i.e., when [X_*i*_]_tot_* is higher), their importance can be even higher. The spatiotemporal variations in the importance of HCOOH and CH_3_COOH buffering, as well as the buffering of other organic acids, need to be clarified with more observations.

## 5 Summary and Future Studies

The carbon dioxide, ammonia, and organic acids show distinct contributions in buffering the acidity of aerosols and clouds. This is mainly due to the large shifts in their multiphase buffering pH ranges, considering the much higher liquid water contents of clouds than aerosols. For CO_2_/HCO_3_^–^, its p*K*_a_* for aerosols is about 15.8–18.4, which is too far away from the typical aerosol pH ranges of <7, and therefore, its buffering capacity is negligible. In comparison, for clouds, the p*K*_a_* of CO_2_/HCO_3_^–^ would decrease to around 11–13, and the corresponding *b*_i_* can be compensated by its high abundances when the cloud pH is higher. For ammonia, its p*K*_a_* varied just in the right range (0–5) for aerosols and is usually the dominant buffering species for large parts of the continental urban areas. For clouds, the p*K*_a_* of ammonia increased to ∼7, and its contribution to the cloud buffering depends on the actual cloud pH. As for organic acids, most *n*-alkanoic monocarboxylic acids are unlikely to buffer the aerosols due to the too high p*K*_a_* values. While the C2–C9 aliphatic dicarboxylic acids and some other acids are with the right p*K*_a,i_* values, their contribution to aerosol buffering is often overwhelmed by that of ammonia. In clouds, however, most of the monocarboxylic acids have the proper p*K*_a_*. Combined with the typically higher abundances (especially HCOOH and CH_3_COOH), their contribution to cloud buffering can be important. Note that in clouds and rains, despite the 10^4^ to 10^7^ higher abundance of CO_2_, its buffering effect is only comparable with that of ammonia and organic acids due to the large pH–p*K*_a_* gaps of CO_2_. Therefore, the buffering effect of CO_2_ can be readily overcome by the acidification of trace acidic gases such as SO_2_ or NO_x_, which would result in acid rain.

Despite the progress made in the potential role and major influencing factors of atmospheric weak acids and bases in regulating the acidity of atmospheric water, substantial uncertainties remain in the quantified estimation of their importance. For a deeper and more quantified understanding, we propose that future studies should focus first on the following aspects.

### Identifying Key Organic Acids and the Comprehensive Representation of Their Thermodynamic Properties

Currently, the representation of the fundamental thermodynamic properties of organic acids is insufficient. For example, the temperature dependences of *K*_a_ and *H*_*i*_ of many organic acids are lacking,^74,75^ which can cause rather large estimation uncertainties for clouds or during winters of the temperate zone, where the temperature are usually below 0 °C. However, considering the wide varieties of organic acids, it can be quite time consuming and technically challenging to obtain all relevant thermodynamic properties experimentally for all species, even considering the advances in theoretical calculations (e.g., refs (81 and 82)). Therefore, further studies are needed to identify the most important species and the major influencing factors of their properties under different conditions and thus to give the simplified and representative scenario-specific parametrizations.

As illustrated by the discussions above, the important organic acids in influencing the system buffering should meet the following criteria. First, they need to be with enough abundances (i.e., relatively high [X_i_]_tot_). Second, p*K*_a_* values need to be within the typical pH range of aerosols/clouds so that *b*_i_ is not too small. Third, p*K*_a_* values should differ with that of ammonia at the given *L*_w_ and temperature conditions in that region/periods; otherwise, the ammonia buffering would be totally overwhelming. Especially, for most continental aerosols, the ammonia buffering is so strong that the organic acids often play only a negligible or minor role (Section 4.2). Under such conditions, the influence of nonideality, etc. can be more important than that of organic acids. In clouds, however, the p*K*_a_* of ammonia is relatively high (Figure 4), and buffering of organic acids can be important.

### More Sophisticated Chemical Spectrum Observations

Currently, the measurements of chemical compositions including both inorganic species and organic acids in both gas and particle phases are scarce, especially for fogs and clouds. In addition, the pH of individual cloud drops can vary with drop sizes, etc. within a given cloud,^49,83−85^ while the size-dependent measurements are also rare. As the contribution of organic acids to cloud acidity can be quite important, we encourage such campaigns in the future.

### Influence of Nonideality in Aerosol Water

The deliquescent aerosols are highly nonideal, with high ionic strength up to ∼43 mol kg^–1^ in severe urban hazes.^8^ This kind of high nonideality can shift the p*K*_a_* of ammonia by up to ∼1 unit.^30,36^ Moreover, the influence of nonideality depends not only on the ionic strength, but also on the aerosol composition and the specific ion pairs.^36^ Different thermodynamic models disagree with each other even for the nonideality for inorganic species,^5,86,87^ not to mention the organic acids. As discussed in Section 4.2, while the potentially buffering organic acids are usually with much lower abundances than that of ammonia, they may contribute certain buffering effects when their buffering ranges differ much with that of ammonia. At a given aerosol water content, the nonideality may either narrow or broaden the p*K*_a_* gaps between ammonia and organic acids and therefore enhance or weaken the contribution of organic acids in buffering the aerosol pH.

### Interactions among Organic Acids, Cloud Acidity, and Cloud Chemistry

As discussed in Section 4.3, the organic acids, especially HCOOH and CH_3_COOH, can potentially exert strong buffering effects in fogs and clouds. On the other hand, in-cloud reactions are shown to be an important source of organic acids,^63,88,89^ while the efficiency of which can be susceptible to acidity.^2^ In addition, unlike aerosols, the gas–liquid equilibrium times for bigger droplets like clouds are longer, and the time scales may differ between the buffering effects and the in-cloud reactions. The feedback among these processes under different conditions needs further exploring.

## Supporting Information Available

SI Text S1 and Table S1: Additional discussion of the dependence of *b*_*i*_ on |pH–p*K*_a,*i*_|. SI Text S2 and Figure S2: Case studies on the modification of cloud pH by atmospheric acids/bases. Figure S1: Additional information on the dependence of p*K*_a_* on temperature. Figure S3: Dependence of the difference between *K*_a_* and *K*_a_ on liquid water content *L*_w_ and Henry’s constant *H*_*i*_. Table S2: Detailed scenario settings as shown in Figure 2b and Figure 3. Table S3: Acid dissociation constant *K*_a_ and Henry’s constant *H*_*i*_ of some commonly observed low molecular weight organic acids in the atmosphere (PDF)
