# Supplementary material for: Role of Carbon Dioxide, Ammonia, and Organic Acids in Buffering Atmospheric Acidity: The Distinct Contribution in Clouds and Aerosols
Source: Environ Sci Technol. 2023 Aug 21;57(34):12571–82. doi: 10.1021/acs.est.2c09851 (PMC10469486; doi:10.1021/acs.est.2c09851)
Supplement: Supplementary file 1 — es2c09851_si_001.pdf [file es2c09851_si_001.pdf]

Supporting Information for

Role of Carbon Dioxide, Ammonia, and Organic Acids in Buffering Atmospheric Acidity: The Distinct Contribution in Clouds and Aerosols

Guangjie Zheng[1,3*], *Hang Su*[2,4], *Yafang Cheng*[1*]

[1] Minerva Research Group, Max Planck Institute for Chemistry, Mainz 55128, Germany

[2] Multiphase Chemistry Department, Max Planck Institute for Chemistry, Mainz 55128, Germany

[3] State Key Joint Laboratory of Environmental Simulation and Pollution Control, School of Environment, Tsinghua University, Beijing 100084, China

[4] Institute of Atmospheric Physics, Chinese Academy of Sciences, Beijing, 100029, China

* Correspondence to: Y.C (yafang.cheng@mpic.de) and G.Z (zgj123@mail.tsinghua.edu.cn)

This 4-page PDF file contains:

- SI Text S1-S2
- Figure S1-S3
- Table S1-S3
- Additional References

S1: Dependence of b_i on |pH- p$K_{a,i}$|

Based on the definition of b_i, we have:

$$b_i = \frac{K_{a,i}[H^+(aq)]}{(K_{a,i}+[H^+(aq)])^2} = \frac{K_{a,i}^2 \frac{[H^+(aq)]}{K_{a,i}}}{K_{a,i}^2(1+\frac{[H^+(aq)]}{K_{a,i}})^2} = \frac{\frac{[H^+(aq)]}{K_{a,i}}}{(1+\frac{[H^+(aq)]}{K_{a,i}})^2}$$

Let x = $[H^+]/K_{a,i}$, then $x^{-1} = K_{a,i}/[H^+]$, and it's easy to prove that:

$$b_i = \frac{x}{(1+x)^2} = \frac{x^{-1}}{(1+x^{-1})^2}$$

That is, b_i is a function of $[H^+]/K_{a,i}$, or equivalently $K_{a,i}/[H^+]$. As x or x^{-1} can be very large or small numbers, it's easier to express them in the logistics way. That is, b_i depends on |logx| = $|\log(x^{-1})|$ = |pH - p$K_{a,i}$|.

S2: Case studies on the modification of cloud pH by atmospheric acids / bases

Take the cloud scenarios as shown in Fig. S2 for example. In sharp contrast with the aerosol systems (Fig. 1a), the cloud systems are easily acidified by the uptake of trace amount of atmospheric acids. For example, with the uptake of ~2.5 ppb of strong acids (e.g., HCl) by the cloud droplets, the cloud pH would drop from 5.6 to 3 for the system with L_w of 0.1 g m^{-3} (Fig. S2a), and drop to 4 when L_w is 1 g m^{-3} (Fig. S2b). For the system with L_w of 0.1 g m^{-3} (Fig. S2a), the pH response curve is nearly the same with the pure water droplets in the typical cloud pH ranges of 2-7, indicating negligible buffering effect of CO_2. For the system with higher L_w of 1 g m^{-3} (Fig. S2b), the pH response curves began to differ when pH is over ~5 due to the buffering effect of CO_2. However, this buffering effect is quite limited, and the cloud pH would increase from 5.6 to 7 with the uptake of only ~1.3 ppb of strong base.

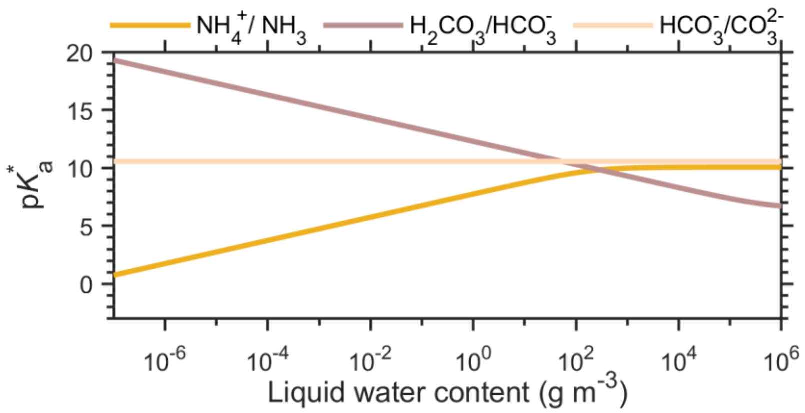

Figure S1. Variation of the pK_a^* of H_2CO_3/HCO_3^-, HCO_3^-/CO_3^{2-} in comparison with that of the NH_4^+/NH_3 with liquid water content L_w at 273 K.

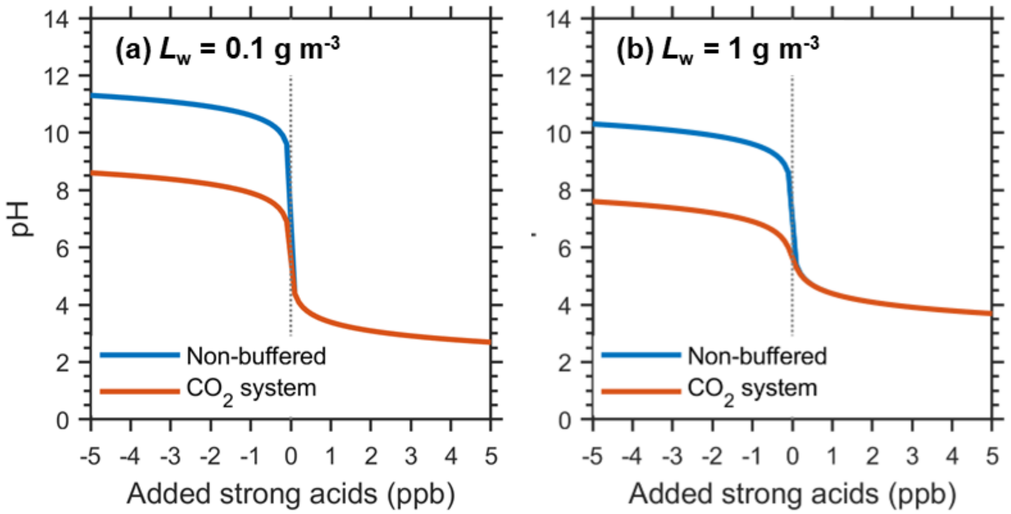

Figure S2. Modification of cloud pH by trace amount of atmospheric acids / bases. The non-buffered system represents pure water droplets, while the CO_2 system represents the real ambient conditions when the water droplets are in equilibrium with 350 ppm of CO_2. Here the added strong acids are shown in atmospheric volume mixing ratios to be comparable with ambient conditions. Note here the strong acids are the equivalent strong monoacids (e.g., HCl) uptake by the cloud droplets, while the negative values indicate the uptake amount of equivalent strong mono-bases.

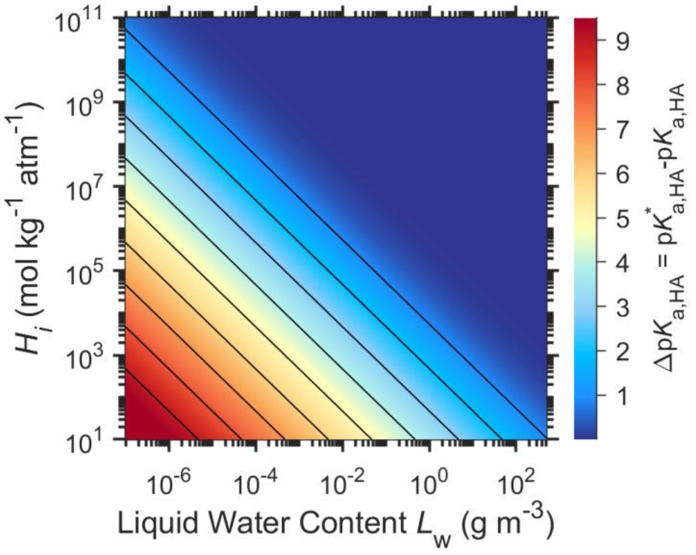

Figure S3. Dependence of the difference between K_a^* and K_a on liquid water content L_w and Henry's constant H_i at 298 K.

Table S1. Dependence of b_i on the difference between pH and $pK_{a,i}$. Read 1.0E-3 as 1.0×10^{-3}.

$\lvert pH-pK_{a,i} \rvert$	0	1	2	3	4	5	6	7	8	9	10
b_i	2.5E-1	8.3E-2	9.8E-3	1.0E-3	1.0E-4	1.0E-5	1.0E-6	1.0E-7	1.0E-8	1.0E-9	1.0E-10

Table S2. Scenario settings as shown in Fig. 2b and Fig. 3. The total (gas and particle phase) concentration of all species shown are in the unit of μmol/m^3.

Scenario	SE-US Fall	Beijing	Polluted Fog	Cloud [d]
L_w (g m^{-3})	2.2×10^{-6}	8.0×10^{-5}	0.094 [b]	0.37
T (K)	298.0	269.0	277.0 [b]	272.7
Total sulfate (μmol/m^3)	0.016	0.370	0.145 [b]	0.003
Total NH_3 (μmol/m^3)	0.364	2.309	0.521 [b]	0.030
Total HNO_3 (μmol/m^3)	0.011	0.315	0.057 [b]	0.011
Total HCOOH (μmol/m^3)	0.049	0.108	0.078 [c]	0.007
Total CH_3COOH (μmol/m^3)	0.033	0.041	0.145 [c]	0.012
Total $(COOH)_2$ (μmol/m^3)	0.011[a]	0.053[a]	0.015 [c]	4.60×10^{-4}
CO_2 (ppm)	410	410	410	410
Ref.	[1]	[2]	[3-5]	[6]

[a] Here the concentration is amplified by 10 times to provide an upper limit of all potential buffering organic acids.

[b] Averages of the reported data for the fog event at Bakersfield during 0:00-4:00 PST, Jan. 13, 1984 in ref. [3-4].

[c] Estimated by the sulfate concentration (i.e., 0.145 μmol/m^3) and the ratios of corresponding organic acids to sulfate as reported in Table 1 of ref. [5].

[d] Here the cloud event #1 in ref. [6] is used.

Table S3. The acid dissociation constant, K_a, at 25 °C and Henry's constant, H_i, of some commonly observed low molecular weight organic acids in the atmosphere. The value of K_a are based on ref. [74] while that of H_i are based on ref. [75] and the references therein unless otherwise noted. The "Abbr" refer to the abbreviations shown in Fig. 3, which include C1-C9 *n*-alkanoic monocarboxylic acids (mC1-mC9), C2-C9 aliphatic dicarboxylic acids (dC2-dC9), and other acids. For polyacids, only the dissociation of the 1st carboxyl group is considered here.

Category	Name	Abbr.	pK_a	H_i Emol kg^{-1} atm^{-1} [a]	dlnH_i/d(1/T) (K)	Potential aerosol buffers[c]	Potential cloud buffers[d]
n-alkanoic monocarboxylic acids	Formic acid	mC1	3.75	8.92E3	6100	Yes	Yes
	Acetic acid	mC2	4.756	4.05E3	6200		Yes
	Propionic acid	mC3	4.87	1.52E3	6800		Yes
	Butyric acid	mC4	4.83	9.83E2	7100		Yes
	Valeric acid	mC5	4.83	2.33E3	6900		Yes
	Caproic acid	mC6	4.85	1.32E3	6100		Yes
	Enanthic acid	mC7	4.89	9.73E2	8500		Yes
	Caprylic acid	mC8	4.89	1.52E1	9600		
	Pelargonic acid	mC9	4.96	3.85E2			Yes
aliphatic dicarboxylic acids	Oxalic acid	dC2	1.25	7.19E8	9800	Yes	
	Malonic acid	dC3	2.85	3.85E10	11000	Yes	
	Succinic acid	dC4	4.21	4.15E9	11000	Yes	Yes
	Glutaric acid	dC5	4.32	5.17E9	12000	Yes	Yes
	Adipic acid	dC6	4.41	6.69E9	13000	Yes	Yes
	Pimelic acid	dC7	4.71	8.21E9	15000	Yes	Yes
	Suberic acid	dC8	4.526	7.80E9	14000	Yes	Yes
	Azelaic acid	dC9	4.53	9.02E9	17000	Yes	Yes
	Sebacic acid	dC10	4.59	7.70E9		Yes	Yes
Aromatic carboxylic acid	Benzoic acid	C7H6O2	4.20	1.42E4	6500	Yes	Yes
	Phenylacetic acid	C8H8O2	4.31	1.52E4			Yes
	Phthalic acid	C8H6O4	2.943	4.96E7		Yes	
Other	Glyoxylic acid	C2H2O3	3.18	1.11E4	4800	Yes	Yes
	Glycolic acid	C2H4O3	3.83	2.84E4	4000	Yes	Yes
	Acrylic acid	C3H4O2	4.25	3.14E3			Yes
	Pyruvic acid	C3H4O3	2.39	3.14E5	5100	Yes	
	Lactic acid	C3H6O3	3.86	1.22E4		Yes	Yes
	Maleic acid	C4H4O4	1.92	1.42E10		Yes	
	Fumaric acid	C4H4O4	3.02	1.42E10[b]		Yes	Yes
	Malic acid	C4H6O5	3.40	2.74E10		Yes	Yes
	DL-Tartaric acid	C4H6O6	3.03	1.00E18		Yes	Yes
	Citric acid	C6H8O7	3.13	3.04E18		Yes	Yes

[a] Read 1.02E3 as 1.02 $\times 10^3$.

[b] Assumed to be the same as maleic acid.

[c] Those with the minimum $pK_a^* < 7$ in the typical aerosol water ranges of 10^{-6} - 5×10^{-3} g m^{-3}.

[d] Those with the pK_a^* of 3 - 7 in the typical liquid water content ranges for clouds of 0.05 to 3 g m^{-3}.

REFERENCES

1. Nah, T.; Guo, H.; Sullivan, A. P.; Chen, Y.; Tanner, D. J.; Nenes, A.; Russell, A.; Ng, N. L.; Huey, L. G.; Weber, R. J., Characterization of aerosol composition, aerosol acidity, and organic acid partitioning at an agriculturally intensive rural southeastern US site. *Atmos. Chem. Phys.* **2018,** *18* (15), 11471-11491.

2. Wang, Y.; Zhuang, G.; Chen, S.; An, Z.; Zheng, A., Characteristics and sources of formic, acetic and oxalic acids in PM2.5 and PM10 aerosols in Beijing, China. *Atmospheric Research* **2007,** *84* (2), 169-181.

3. Jacob, D. J. The Origins of Inorganic Acidity in Fogs. Dissertation (Ph.D.). California Institute of Technology, 1985.

4. Jacob, D. J.; Waldman, J. M.; Munger, J. W.; Hoffmann, M. R., The H2SO4-HNO3-NH3 system at high humidities and in fogs: 2. Comparison of field data with thermodynamic calculations. *Journal of Geophysical Research: Atmospheres* **1986,** *91* (D1), 1089-1096.

5. Collett, J. L.; Hoag, K. J.; Sherman, D. E.; Bator, A.; Richards, L. W., Spatial and temporal variations in San Joaquin Valley fog chemistry. *Atmospheric Environment* **1998,** *33* (1), 129-140.

6. Sellegri, K.; Laj, P.; Marinoni, A.; Dupuy, R.; Legrand, M.; Preunkert, S., Contribution of gaseous and particulate species to droplet solute composition at the Puy de Dôme, France. *Atmos. Chem. Phys.* **2003,** *3* (5), 1509-1522.
